# The 1:1 cocrystal of *rac*-7-oxabicyclo­[2.2.1]heptane-2,3-dicarboxylic acid and 2-amino­benzothia­zole

**DOI:** 10.1107/S1600536808015365

**Published:** 2008-07-09

**Authors:** Yun-Yun Wang, Rui-Ding Hu, Yan-Jun Wang

**Affiliations:** a Zhejiang Key Laboratory for Reactive Chemistry on Solid Surfaces, Institute of Physical Chemistry, Zhejiang Normal University, Jinhua, Zhejiang 321004, People’s Republic of China, and College of Chemistry and Life Sciences, Zhejiang Normal University, Jinhua 321004, Zhejiang, People’s Republic of China

## Abstract

In the crystal structure of the title compound, *rac*-7-oxabicyclo­[2.2.1]heptane-2,3-dicarboxylic acid–2-amino­benzo­thia­zole (1/1), C_8_H_10_O_5_·C_7_H_6_N_2_S, mol­ecules of each component are linked into centrosymmetric dimers by inter­molecular N—H⋯O hydrogen bonds. These dimers are connected by O—H⋯O hydrogen bonds into a chain along the *b* axis. In addition, π–π inter­actions between aromatic heterocycles occur [centroid–centroid distance of 3.4709 Å and inter­planar spacing of 3.4374 Å between symmetry-related benzothia­zole ring systems.

## Related literature

For related literature, see: Liu *et al.* (2002 ▶).
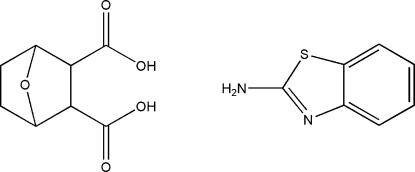


## Experimental

### 

#### Crystal data

C_8_H_10_O_5_·C_7_H_6_N_2_S
*M* *_r_* = 336.36
Triclinic, 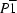

*a* = 8.3082 (1) Å
*b* = 9.0428 (1) Å
*c* = 11.0438 (2) Å
α = 67.1546 (8)°
β = 83.0101 (8)°
γ = 86.9193 (9)°
*V* = 758.92 (2) Å^3^
*Z* = 2
Mo *K*α radiation
μ = 0.24 mm^−1^
*T* = 296 (2) K
0.43 × 0.27 × 0.16 mm

#### Data collection

Bruker APEXII area-detector diffractometer
Absorption correction: multi-scan (*SADABS*; Sheldrick, 1996 ▶) *T*_min_ = 0.92, *T*_max_ = 0.96
11829 measured reflections
3416 independent reflections
2675 reflections with *I* > 2σ(*I*)
*R*_int_ = 0.026

#### Refinement

*R*[*F* ^2^ > 2σ(*F* ^2^)] = 0.056
*wR*(*F* ^2^) = 0.159
*S* = 1.06
3416 reflections
214 parameters
4 restraints
H atoms treated by a mixture of independent and constrained refinement
Δρ_max_ = 0.67 e Å^−3^
Δρ_min_ = −1.05 e Å^−3^

### 

Data collection: *SMART* (Bruker, 2004 ▶); cell refinement: *SAINT* (Bruker, 2004 ▶); data reduction: *SAINT*; program(s) used to solve structure: *SHELXS97* (Sheldrick, 2008 ▶); program(s) used to refine structure: *SHELXL97* (Sheldrick, 2008 ▶); molecular graphics: *SHELXTL* (Sheldrick, 2008 ▶); software used to prepare material for publication: *SHELXL97*.

## Supplementary Material

Crystal structure: contains datablocks I, global. DOI: 10.1107/S1600536808015365/kp2168sup1.cif

Structure factors: contains datablocks I. DOI: 10.1107/S1600536808015365/kp2168Isup2.hkl

Additional supplementary materials:  crystallographic information; 3D view; checkCIF report

## Figures and Tables

**Table 1:** Hydrogen-bond geometry (Å, °)

*D*—H⋯*A*	*D*—H	H⋯*A*	*D*⋯*A*	*D*—H⋯*A*
N1—H1*A*⋯O1^i^	0.86	2.13	2.953 (3)	161
N1—H1*B*⋯O5	0.86	2.29	3.061 (3)	150
N1—H1*B*⋯O4	0.86	2.38	2.991 (3)	128
O2—H2⋯O4^ii^	0.836 (18)	1.868 (18)	2.700 (2)	174 (3)
O3—H3⋯N2^i^	0.854 (18)	1.758 (19)	2.611 (2)	176 (4)

Symmetry codes: (i) 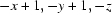; (ii) 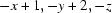.

**Fig. 1.**
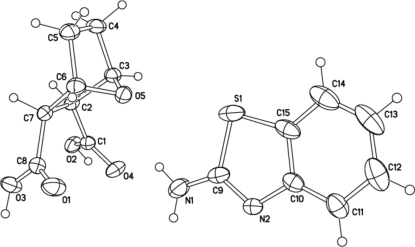
A view of the molecule of (I) showing the atom-labelling scheme with displacement ellipsoids drawn at the 30% probability.

**Fig. 2.**
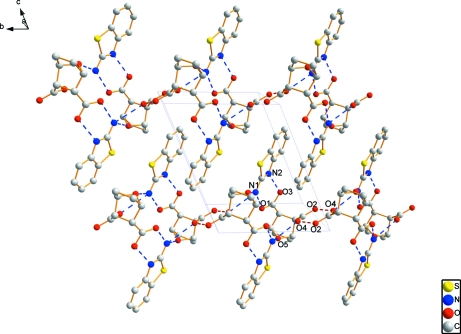
The crystal packing diagram, showing the π···π stacking and hydrogen bonds as dash lines.

## supplementary crystallographic information

### Comment

7-Oxabicyclo[2.2.1]heptane-2,3-dicarboxylic anhydride (norcantharidin), a traditional Chinese drug, has great anti-cancer activity. In order to prepare compounds with pronounced anti-cancer activity, some derivatives were synthesized (Liu *et al.*, 2002). 7-oxabicyclo[2.2.1]heptane-2,3-dicarboxylic anhydride can react with 2-aminobenzothiazole to form an acylamide acid derivative which has strong anti-cancer activity. However, a crystal suitable for X-ray diffraction was obtained during the synthesis unexpectedly.

The crystal structure of the title compound (I) is characterized by alternating molecules of 7-oxabicyclo[2.2.1]heptane-2,3-dicarboxylic acid and 2-aminobenzothiazole, linked by N—H···O and O—H···O hydrogen bonds. The centrosymmetric dimer composed of two 2-aminobenzothiazole and two acids is generated by bifurcated hydrogen bonds of amino group of 2-aminobenzothiazole and the acid component (N1—H1B···O4 and N1—H1B···O5, O3—H3···N2 and N1—H1A···O1). These dimers are connected into a chain by hydrogen bonds O2—H2···O4. Furthermore, there are short distances [centroid separation of 3.4709 Å and interplanar spacing of 3.4374 Å] between the benzothiazole-ring planes and the symmetry-related planes at (-*x* + 1,-*y* + 1,-*z*; -*x* + 1,-*y* + 2,-*z*) of adjacent chains, implying π···π interactions (Fig. 2). In the molecule, the conformation of 7-oxabicyclo[2.2.1]heptane ring is discussed as follows, the six-membered ring adopts a boat conformation and the two oxygen-bearing five-membered heterocycles are in an envelope conformation.

### Experimental

7-Oxabicyclo[2.2.1]heptane-2,3-dicarboxylic anhydride and 2-aminobenzothiazole were dissolved in acetonitrile and the mixture was stirred for 2 h at room temperature. The precipitate has been proved to exhibit anticancer activity. However, colourless crystals of (I) were obtained in the filtrate after several days, unexpectedly.

### Refinement

The structure was solved by direct methods and successive Fourier difference synthesis. The H atoms bonded to C and Natoms were positioned geometrically and refined using a riding model [aromatic C—H 0.93 Å, aliphatic C—H = 0.97 (2) Å and N—H = 0.86 Å *U*_iso_(H) = 1.2*U*_eq_(C)]. The H atoms bonded to O atoms were located in a differenceFourier maps and refined with O—H distance restraints of 0.85 (2) and *U*_iso_(H) = 1.5*U*_eq_(O).

### Crystal data

**Table:**

C_8_H_10_O_5_·C_7_H_6_N_2_S	*Z* = 2
*M**_r_* = 336.36	*F*_000_ = 352
Triclinic, *P*1	*D*_x_ = 1.472 Mg m^−^^3^
Hall symbol: -P 1	Mo *K*α radiation λ = 0.71073 Å
*a* = 8.3082 (1) Å	Cell parameters from 4303 reflections
*b* = 9.0428 (1) Å	θ = 2.0–27.4º
*c* = 11.0438 (2) Å	µ = 0.24 mm^−^^1^
α = 67.1546 (8)º	*T* = 296 (2) K
β = 83.0101 (8)º	Block, colourless
γ = 86.9193 (9)º	0.43 × 0.27 × 0.16 mm
*V* = 758.93 (2) Å^3^	

### Data collection

**Table:**

Bruker APEXII area-detector diffractometer	3416 independent reflections
Radiation source: fine-focus sealed tube	2675 reflections with *I* > 2σ(*I*)
Monochromator: graphite	*R*_int_ = 0.026
*T* = 296(2) K	θ_max_ = 27.4º
ω scans	θ_min_ = 2.0º
Absorption correction: multi-scan(SADABS; Sheldrick, 1996)	*h* = −10→10
*T*_min_ = 0.92, *T*_max_ = 0.96	*k* = −11→11
11829 measured reflections	*l* = −14→14

### Refinement

**Table:**

Refinement on *F*^2^	Secondary atom site location: difference Fourier map
Least-squares matrix: full	Hydrogen site location: inferred from neighbouring sites
*R*[*F*^2^ > 2σ(*F*^2^)] = 0.056	H atoms treated by a mixture of independent and constrained refinement
*wR*(*F*^2^) = 0.159	*w* = 1/[σ^2^(*F*_o_^2^) + (0.0773*P*)^2^ + 0.3994*P*] where *P* = (*F*_o_^2^ + 2*F*_c_^2^)/3
*S* = 1.07	(Δ/σ)_max_ < 0.001
3416 reflections	Δρ_max_ = 0.67 e Å^−^^3^
214 parameters	Δρ_min_ = −1.05 e Å^−^^3^
4 restraints	Extinction correction: none
Primary atom site location: structure-invariant direct methods	

### Special details

**Table:**

Geometry. All e.s.d.'s (except the e.s.d. in the dihedral angle between two l.s. planes) are estimated using the full covariance matrix. The cell e.s.d.'s are taken into account individually in the estimation of e.s.d.'s in distances, angles and torsion angles; correlations between e.s.d.'s in cell parameters are only used when they are defined by crystal symmetry. An approximate (isotropic) treatment of cell e.s.d.'s is used for estimating e.s.d.'s involving l.s. planes.
Refinement. Refinement of *F*^2^ against ALL reflections. The weighted *R*-factor *wR* and goodness of fit *S* are based on *F*^2^, conventional *R*-factors *R* are based on *F*, with *F* set to zero for negative *F*^2^. The threshold expression of *F*^2^ > σ(*F*^2^) is used only for calculating *R*-factors(gt) *etc*. and is not relevant to the choice of reflections for refinement. *R*-factors based on *F*^2^ are statistically about twice as large as those based on *F*, and *R*- factors based on ALL data will be even larger.

### Fractional atomic coordinates and isotropic or equivalent isotropic displacement parameters (Å^2^)

**Table:**

	*x*	*y*	*z*	*U*_iso_*/*U*_eq_	
S1	0.41626 (8)	0.33690 (10)	0.46435 (8)	0.0645 (3)	
N1	0.5173 (3)	0.4819 (3)	0.2070 (3)	0.0640 (6)	
H1A	0.5827	0.4957	0.1369	0.077*	
H1B	0.4338	0.5433	0.2033	0.077*	
N2	0.6705 (2)	0.2670 (2)	0.33434 (19)	0.0424 (4)	
O1	0.2197 (2)	0.5380 (2)	−0.00822 (19)	0.0616 (5)	
O2	0.28892 (19)	1.05947 (19)	−0.01033 (17)	0.0463 (4)	
H2	0.385 (2)	1.090 (4)	−0.025 (3)	0.069*	
O3	0.1725 (2)	0.7920 (2)	−0.13815 (17)	0.0546 (5)	
H3	0.227 (4)	0.770 (4)	−0.200 (3)	0.082*	
O4	0.40832 (17)	0.81995 (19)	0.06576 (18)	0.0489 (4)	
O5	0.17176 (18)	0.58165 (18)	0.27645 (15)	0.0441 (4)	
C1	0.2856 (2)	0.9053 (2)	0.0535 (2)	0.0354 (4)	
C2	0.1220 (2)	0.8368 (2)	0.1213 (2)	0.0342 (4)	
H2A	0.0424	0.9240	0.1093	0.041*	
C3	0.1319 (3)	0.7405 (3)	0.2703 (2)	0.0405 (5)	
H3A	0.2094	0.7840	0.3079	0.049*	
C4	−0.0397 (3)	0.7220 (3)	0.3424 (2)	0.0486 (6)	
H4A	−0.1005	0.8219	0.3116	0.058*	
H4B	−0.0381	0.6847	0.4373	0.058*	
C5	−0.1091 (3)	0.5947 (3)	0.3034 (3)	0.0498 (6)	
H5A	−0.1388	0.4980	0.3802	0.060*	
H5B	−0.2028	0.6353	0.2554	0.060*	
C6	0.0343 (3)	0.5643 (3)	0.2149 (2)	0.0426 (5)	
H6A	0.0305	0.4601	0.2074	0.051*	
C7	0.0545 (2)	0.7055 (3)	0.0818 (2)	0.0368 (5)	
H7A	−0.0530	0.7396	0.0525	0.044*	
C8	0.1598 (3)	0.6691 (3)	−0.0256 (2)	0.0416 (5)	
C9	0.5451 (3)	0.3664 (3)	0.3202 (3)	0.0473 (6)	
C10	0.6687 (3)	0.1569 (3)	0.4631 (2)	0.0460 (5)	
C11	0.7797 (4)	0.0351 (3)	0.5088 (3)	0.0623 (7)	
H11A	0.8695	0.0243	0.4536	0.075*	
C12	0.7536 (5)	−0.0717 (4)	0.6404 (3)	0.0803 (10)	
H12A	0.8273	−0.1547	0.6738	0.096*	
C13	0.6181 (5)	−0.0553 (4)	0.7222 (3)	0.0823 (10)	
H13A	0.6013	−0.1294	0.8089	0.099*	
C14	0.5100 (5)	0.0671 (5)	0.6778 (3)	0.0788 (9)	
H14A	0.4208	0.0778	0.7335	0.095*	
C15	0.5351 (3)	0.1745 (3)	0.5489 (3)	0.0571 (5)	

### Atomic displacement parameters (Å^2^)

**Table:**

	*U*^11^	*U*^22^	*U*^33^	*U*^12^	*U*^13^	*U*^23^
S1	0.0463 (4)	0.0910 (6)	0.0724 (5)	−0.0114 (3)	0.0153 (3)	−0.0539 (4)
N1	0.0493 (12)	0.0656 (14)	0.0688 (16)	0.0157 (10)	−0.0021 (11)	−0.0202 (12)
N2	0.0411 (9)	0.0464 (10)	0.0417 (10)	−0.0012 (8)	0.0021 (8)	−0.0211 (9)
O1	0.0721 (12)	0.0542 (10)	0.0538 (11)	0.0143 (9)	0.0059 (9)	−0.0212 (9)
O2	0.0363 (8)	0.0413 (8)	0.0499 (10)	−0.0046 (6)	0.0004 (7)	−0.0062 (7)
O3	0.0684 (11)	0.0541 (10)	0.0368 (9)	0.0106 (8)	0.0043 (8)	−0.0171 (8)
O4	0.0303 (7)	0.0460 (9)	0.0630 (11)	0.0003 (6)	0.0053 (7)	−0.0159 (8)
O5	0.0379 (8)	0.0474 (9)	0.0401 (9)	0.0018 (6)	−0.0035 (6)	−0.0098 (7)
C1	0.0315 (9)	0.0389 (10)	0.0332 (10)	−0.0018 (8)	0.0010 (8)	−0.0122 (8)
C2	0.0265 (9)	0.0372 (10)	0.0373 (11)	0.0009 (7)	0.0013 (7)	−0.0141 (9)
C3	0.0350 (10)	0.0506 (12)	0.0368 (11)	−0.0064 (9)	0.0012 (8)	−0.0184 (10)
C4	0.0433 (12)	0.0602 (14)	0.0400 (12)	−0.0075 (10)	0.0110 (9)	−0.0205 (11)
C5	0.0397 (12)	0.0591 (14)	0.0452 (13)	−0.0140 (10)	0.0079 (10)	−0.0162 (11)
C6	0.0405 (11)	0.0420 (11)	0.0440 (12)	−0.0060 (9)	0.0013 (9)	−0.0162 (10)
C7	0.0296 (9)	0.0447 (11)	0.0378 (11)	−0.0007 (8)	−0.0028 (8)	−0.0181 (9)
C8	0.0381 (11)	0.0503 (13)	0.0394 (12)	0.0028 (9)	−0.0046 (9)	−0.0210 (10)
C9	0.0367 (11)	0.0551 (13)	0.0583 (15)	−0.0070 (10)	0.0065 (10)	−0.0335 (12)
C10	0.0556 (14)	0.0478 (12)	0.0408 (12)	−0.0131 (10)	−0.0007 (10)	−0.0234 (10)
C11	0.088 (2)	0.0553 (15)	0.0482 (15)	−0.0018 (14)	−0.0140 (14)	−0.0224 (12)
C12	0.129 (3)	0.0528 (16)	0.062 (2)	−0.0111 (18)	−0.034 (2)	−0.0174 (15)
C13	0.125 (3)	0.083 (2)	0.0391 (15)	−0.0536 (16)	−0.0057 (17)	−0.0179 (15)
C14	0.091 (2)	0.104 (2)	0.0495 (17)	−0.0510 (15)	0.0100 (14)	−0.0374 (17)
C15	0.0619 (14)	0.0761 (14)	0.0439 (14)	−0.0314 (9)	0.0091 (9)	−0.0351 (11)

### Geometric parameters (Å, °)

**Table:**

S1—C15	1.734 (3)	C4—C5	1.537 (3)
S1—C9	1.743 (2)	C4—H4A	0.9700
N1—C9	1.318 (4)	C4—H4B	0.9700
N1—H1A	0.8600	C5—C6	1.530 (3)
N1—H1B	0.8600	C5—H5A	0.9700
N2—C9	1.322 (3)	C5—H5B	0.9700
N2—C10	1.382 (3)	C6—C7	1.525 (3)
O1—C8	1.214 (3)	C6—H6A	0.9800
O2—C1	1.295 (3)	C7—C8	1.517 (3)
O2—H2	0.836 (18)	C7—H7A	0.9800
O3—C8	1.303 (3)	C10—C11	1.375 (4)
O3—H3	0.854 (18)	C10—C15	1.413 (3)
O4—C1	1.234 (2)	C11—C12	1.396 (4)
O5—C3	1.434 (3)	C11—H11A	0.9300
O5—C6	1.443 (3)	C12—C13	1.395 (6)
C1—C2	1.508 (3)	C12—H12A	0.9300
C2—C3	1.544 (3)	C13—C14	1.361 (5)
C2—C7	1.565 (3)	C13—H13A	0.9300
C2—H2A	0.9800	C14—C15	1.374 (4)
C3—C4	1.530 (3)	C14—H14A	0.9300
C3—H3A	0.9800		
			
C15—S1—C9	89.18 (12)	O5—C6—C5	102.47 (18)
C9—N1—H1A	120.0	C7—C6—C5	110.16 (19)
C9—N1—H1B	120.0	O5—C6—H6A	113.6
H1A—N1—H1B	120.0	C7—C6—H6A	113.6
C9—N2—C10	111.49 (19)	C5—C6—H6A	113.6
C1—O2—H2	109 (2)	C8—C7—C6	114.11 (18)
C8—O3—H3	112 (2)	C8—C7—C2	115.16 (16)
C3—O5—C6	96.24 (15)	C6—C7—C2	101.23 (17)
O4—C1—O2	123.04 (18)	C8—C7—H7A	108.7
O4—C1—C2	121.43 (18)	C6—C7—H7A	108.7
O2—C1—C2	115.39 (17)	C2—C7—H7A	108.7
C1—C2—C3	110.23 (17)	O1—C8—O3	124.6 (2)
C1—C2—C7	116.64 (16)	O1—C8—C7	123.1 (2)
C3—C2—C7	100.43 (16)	O3—C8—C7	112.29 (18)
C1—C2—H2A	109.7	N1—C9—N2	123.7 (2)
C3—C2—H2A	109.7	N1—C9—S1	121.07 (18)
C7—C2—H2A	109.7	N2—C9—S1	115.2 (2)
O5—C3—C4	103.13 (17)	C11—C10—N2	125.6 (2)
O5—C3—C2	102.91 (16)	C11—C10—C15	120.4 (2)
C4—C3—C2	108.53 (18)	N2—C10—C15	114.0 (2)
O5—C3—H3A	113.7	C10—C11—C12	118.0 (3)
C4—C3—H3A	113.7	C10—C11—H11A	121.0
C2—C3—H3A	113.7	C12—C11—H11A	121.0
C3—C4—C5	101.17 (18)	C11—C12—C13	120.6 (3)
C3—C4—H4A	111.5	C11—C12—H12A	119.7
C5—C4—H4A	111.5	C13—C12—H12A	119.7
C3—C4—H4B	111.5	C14—C13—C12	121.3 (3)
C5—C4—H4B	111.5	C14—C13—H13A	119.3
H4A—C4—H4B	109.4	C12—C13—H13A	119.3
C6—C5—C4	101.63 (17)	C13—C14—C15	118.7 (3)
C6—C5—H5A	111.4	C13—C14—H14A	120.6
C4—C5—H5A	111.4	C15—C14—H14A	120.6
C6—C5—H5B	111.4	C14—C15—C10	120.9 (3)
C4—C5—H5B	111.4	C14—C15—S1	128.9 (3)
H5A—C5—H5B	109.3	C10—C15—S1	110.1 (2)
O5—C6—C7	102.38 (16)		

### Hydrogen-bond geometry (Å, °)

**Table:**

*D*—H···*A*	*D*—H	H···*A*	*D*···*A*	*D*—H···*A*
N1—H1A···O1^i^	0.86	2.13	2.953 (3)	161
N1—H1B···O5	0.86	2.29	3.061 (3)	150
N1—H1B···O4	0.86	2.38	2.991 (3)	128
O2—H2···O4^ii^	0.836 (18)	1.868 (18)	2.700 (2)	174 (3)
O3—H3···N2^i^	0.854 (18)	1.758 (19)	2.611 (2)	176 (4)

Symmetry codes: (i) −*x*+1, −*y*+1, −*z*; (ii) −*x*+1, −*y*+2, −*z*.
